# Challenges and Opportunities of the Human-Centered Design Approach: Case Study Development of an Assistive Device for the Navigation of Persons With Visual Impairment

**DOI:** 10.2196/70694

**Published:** 2025-08-18

**Authors:** Mario Andres Chavarria, Luisa María Ortiz-Escobar, Bladimir Bacca-Cortes, Victor Romero-Cano, Isabella Villota, Jhon Kevin Muñoz Peña, José Roberto Londoño Sánchez, Oscar Campo, Silvan Suter, Jhon Jairo Cabrera-López, Maria Fernanda Sanchez Patiño, Eduardo Francisco Caicedo-Bravo, Michael Stein, Samia Hurst, Klaus Schönenberger, Minerva Rivas Velarde

**Affiliations:** 1 EssentialTech Centre École Polytechnique Fédérale de Lausanne Lausanne Switzerland; 2 Grupo de Investigación en Ingeniería Biomédica, GBIO Universidad Autónoma de Occidente Cali Colombia; 3 Geneva School of Health Science University of Applied Sciences and Arts Western Switzerland Geneva Switzerland; 4 Institute of Ethics, History, and Humanities University of Geneva Geneva Switzerland; 5 School of Electrical and Electronics Engineering Universidad del Valle Cali Colombia; 6 School of Computer Science and Informatics Cardiff University Cardiff United Kingdom; 7 Hospital Universitario del Valle Cali Colombia; 8 Harvard Law School Project on Disability Harvard University Cambridge, MA United States

**Keywords:** artificial intelligence, assistive technology, blindness, context, disability, electronic travel aid, human-centered design, navigation, low and middle-income countries, LMICs, user-centered design, visual impairment

## Abstract

**Background:**

Visual impairment (VI) significantly impacts quality of life, particularly in autonomous pedestrian navigation. Limitations in independent navigation lead to frustration, diminished confidence, and risks to bodily integrity for individuals with VI. In Colombia, the pilot country of this study, approximately 2 million people live with some form of visual disability. Globally, only 1 in 10 people requiring assistive devices have access to them, with factors such as deficient product design stemming from limited knowledge of user expectations, local needs, and environmental constraints, posing significant challenges, particularly in low- and middle-income countries.

**Objective:**

We aimed to evaluate the feasibility and limitations of applying the human-centered design (HCD) principles outlined by the International Organization for Standardization (ISO) 9241-210:2019 standard in assistive technology (AT) development for individuals with VI in Colombia.

**Methods:**

We developed a prototype navigation device using the HCD principles, emphasizing a thorough analysis of user needs and environmental contexts. The project leveraged multidisciplinary collaboration to address challenges associated with user engagement and design adaptability while managing legal and bureaucratic constraints. The navigation system integrates artificial intelligence algorithms, specifically developed by the research team as part of this work, to enhance its adaptability and responsiveness to diverse environments. The development process featured iterative prototyping cycles, incorporating user feedback at each stage, all within the boundaries of applicable regulatory frameworks.

**Results:**

The development and evaluation of the initial prototype highlighted both the feasibility and key limitations of applying the ISO 9241-210:2019 HCD principles in AT for individuals with VI in the Colombian context. The prototype met several user-defined expectations by prioritizing affordability; extended battery life; autonomy in internet-constrained environments; and improved ergonomics, concealability, aesthetics, and obstacle detection. These achievements demonstrated the potential of HCD to guide context-sensitive innovation. However, the process also revealed significant barriers: limited legal and procedural clarity for engaging users in design phases, difficulties navigating ethics committees, and a lack of practical guidance within the ISO standard itself. These constraints, compounded by challenges in interdisciplinary collaboration, limited the depth and adaptability of user involvement across development stages.

**Conclusions:**

Implementing HCD principles in AT development shows promise for creating effective and affordable solutions tailored to user needs and contexts. However, legislative and methodological barriers must be addressed to fully realize HCD’s potential. Future efforts should focus on aligning research methodologies with hardware and software development practices while integrating legislative frameworks to enhance the accessibility and effectiveness of AT innovations.

## Introduction

### Background

Globally, over 1 billion people live with disabilities, with approximately 80% residing in low- and middle-income countries (LMICs) [1,2]. Disabilities are highly diverse, and individuals benefit from different types of technologies depending on their specific impairments. This paper focuses on pedestrian navigation technologies for individuals who are blind or have disabling low vision. Worldwide, there are 39 million persons who are blind and 246 million persons with moderate to severe visual impairment (VI) [3,4].

Persons with disabilities worldwide face a significantly higher risk of poverty compared to the general population [5]. For many, assistive technologies (ATs) are essential to performing daily activities that would otherwise be difficult or impossible. However, traditional approaches to designing such technologies often fail to reflect the lived realities of those in low-income communities. According to the World Health Organization, only 1 in 10 people in need of assistive products actually have access to them, leaving over 200 million individuals with VIs worldwide without access to assistive products [6]. This lack of access severely restricts their autonomy, social participation, and opportunities for education and employment, further reinforcing cycles of exclusion and inequality.

### AT in the Context of LMICs

Technologies designed for high-income countries often fail to meet their intended impact when applied in LMICs [7,8]. This is particularly evident among individuals with disabilities, where AT devices, whether donated or purchased, face limited adoption, and even when adopted, they tend to have a short life span, that is, access to AT in LMICs can be as low as 3% [6,9]. Inadequate comprehension of the real-life experiences and daily challenges faced by individuals with disabilities in LMICs, coupled with limited awareness of infrastructure constraints and the implications of high maintenance costs, contributes to their exclusion. This hampers the development of sustainable AT solutions and limits the ability of individuals with disabilities in LMICs to lead fulfilling lives. It often begins early in the design process and can permeate the entire development chain, ultimately affecting deployment strategies and business models.

### Existing Technology for Persons With VI in the Market

The simplest and most affordable electronic travel aids (ETA) for individuals with VI are ultrasonic canes and glasses. These systems use ultrasonic sensors to detect obstacles and alert the user with an audible signal. More sophisticated ETA systems provide users with detailed information about their surroundings. One of the earliest and best-known ETA models is the voice navigation system, which uses a camera to monitor the user’s surroundings and uses an algorithm to process the data, converting it into a time-multiplexed auditory representation. Not all ETAs use audio signals; some use tactile interfaces, such as the AuxDeco system’s front-facing display [10], or electronic signals, such as the BrainPort Vision Pro’s tongue display [11]. A recent entry in the ETAs market is Biped [12], a system comprising a large harness that users must wear visibly on their shoulders, equipped with cameras and other electronic components that implement machine learning methods to assist users with VI in detecting obstacles and receiving GPS instructions. Unfortunately, more advanced systems come with significantly higher costs; the prices of most advanced ETAs range in thousands or even tens of thousands of US dollars (eg, AuxDeco: US $10,000 to US $15,000, BrainPort Vision Pro: US $8000 to US $10,000, and BIPED: US $5000).

With the widespread use of smartphones among individuals with VI, navigation such as RightHear [13] and Lazarillo [14] have become popular and affordable solutions for navigating urban environments. However, these only provide map or GPS information and lack real-time details about the surrounding area, such as collision warnings. In addition, they do not function in all environments, especially where GPS is unavailable, such as indoor spaces and rural areas.

### Available Innovation

In recent years, significant research activity has focused on developing technological solutions to assist users with VI in outdoor pedestrian navigation. Despite notable advancements, current systems still fall short of providing comprehensive and robust support that fully meets the navigation needs of users with VI [15-18]. As the technology landscape evolves, addressing the unique challenges faced by these users remains a critical priority.

A key limitation of current systems is their narrow scope in obstacle avoidance [17,18]. Despite a considerable amount of published research on obstacle avoidance, there remains a need for datasets representing the diverse obstacles encountered by users with VI. Current approaches often address only a subset of these challenges. More refined systems are needed to evaluate the level of hazard posed by obstacles and deliver appropriate instructions to the user [17-20].

In addition, most available navigation aids rely on audio feedback, typically through headsets, which may block important environmental sounds [15,18,21]. This issue can be mitigated using bone-conducting headphones, allowing users to stay aware of their surroundings [18,21].

Wearable navigation devices, although still rare, offer critical advantages [15-18,22]. The wearability of such devices is key, with options ranging from waist belts and glasses to handheld smartphones or a hybrid of both. Wearable devices are generally preferred as they free the user’s hands and provide more stable image capture, making them a more practical and user-friendly choice for VI navigation.

In Multimedia Appendix 1 [23-38], we provide a comparison table summarizing the main characteristics of the analyzed related works, including the system developed in this study for reference. The table considers aspects such as indoor and outdoor use; night and day operation; short, medium, and large range operation; dynamic obstacle detection; the sensors used; the computation platform; object recognition capabilities (unevenness, low obstacles, high obstacles, and holes); and the data processing algorithm used. All of the works reviewed can deal with indoor environments, but not with outdoor environments [23-25,30,31,33]. Another environment-related property is that not all works reviewed are suitable for working in night conditions. Regarding the range of operation, most of the systems work up to medium range, but a few of them can work at large ranges [26,31]. Detecting and warning about static and dynamic obstacles is an important feature. However, it is very difficult to deal with dynamic obstacles properly, as shown in several studies [23,24,26,27,29-31].

In general, the most used sensor is the camera (RGBD [red, green, and blue, plus depth], monocular, or stereo). While cameras can be combined with other sensors, doing so imposes significant demands on the computing platform used, which, in most cases, corresponds to an embedded system. Some works [27,29,31] used smartphones, but this resulted in compromised computational power and limited access to additional features. Most of the reviewed works focus on detecting low-level objects. However, some [25,26,29,30] are able to detect both low and head-level obstacles. While navigation capabilities are increasingly gaining attention, integrating them with both low- and head-level obstacle detection is challenging, especially when user feedback is limited to audio alerts.

### Context Matters in AT Design

Access to AT in LMICs can be as low as 3% [1]. The challenges in these contexts differ significantly from high-income countries, hindering AT implementation and adoption [2-4,9]. A primary barrier is cost—many AT devices are prohibitively expensive, with high import duties and maintenance costs further limiting affordability [2,5]. In addition, a lack of qualified personnel often results in misuse and premature equipment failure [2,6].

Beyond financial constraints, infrastructural and environmental factors exacerbate accessibility issues. Unreliable electricity, poor roads, limited water supply, and inaccessible architecture pose major obstacles [2,3,5]. Internet connectivity, essential for many modern ATs, remains scarce, creating a digital divide that limits access to even low-cost or free solutions [5,7]. Harsh environmental conditions, such as high temperatures, humidity, and dust, also shorten the life span of many AT products [6].

Market unpredictability in LMICs presents further challenges, with significant income disparities affecting sustainability. Successful AT deployment requires a deep understanding of local economic and cultural dynamics to ensure long-term adoption and availability [2]. Aesthetic considerations, user expectations, and safety concerns also play a critical role—people in high-crime areas often avoid using high-tech wearable ATs due to the risk of theft. Other common barriers include complexity, discomfort, and sensory overload, which contribute to low adoption rates [5,8].

This work focused on evaluating the implementation of a human-centered design (HCD) approach to enhance the development of an ETA by addressing limitations of the current literature, which include the identified barriers, contextual factors, and user expectations in Colombia (the pilot country). The resulting ETA prototype addressed the limitations of existing technologies by prioritizing cost efficiency, extended battery life, and autonomy in internet-constrained environments. Furthermore, our design enhanced comfort, aesthetics, concealability, and the accuracy of obstacle detection, specifically tailored to the local context.

### Available Guidelines and Frameworks

User-centered design (UCD) approaches have become increasingly prominent in AT development in the past decade [39,40]. UCD has evolved as a design paradigm over the years, giving rise to various methodologies, such as user-centered, human-centered, and context-centered design, with the International Organization for Standardization (ISO) 9241-210:2019 standard for HCD being among the most widely recognized. The ISO 9241-210 standard [41] outlines the principles and guidelines for the HCD process throughout the design and development of interactive systems, with a focus on ensuring usability and user satisfaction. The key principles and activities of the HCD process are structured into different general phases, as described in Textbox 1.

**Phases of the human-centered design process.**

- Phase 1: “Understand and specify the context of use”—involves defining user characteristics, tasks, and goals, as well as understanding the conditions in which the product will be used.
- Phase 2: “Specify the user requirements”—focuses on comprehending user needs and expectations and establishing usability and performance criteria.
- Phase 3: “Produce design solutions”—entails the generation and refinement of design alternatives.
- Phase 4: “Evaluate designs against requirements”—involves usability testing and feedback assessment.

**Figure 1:**
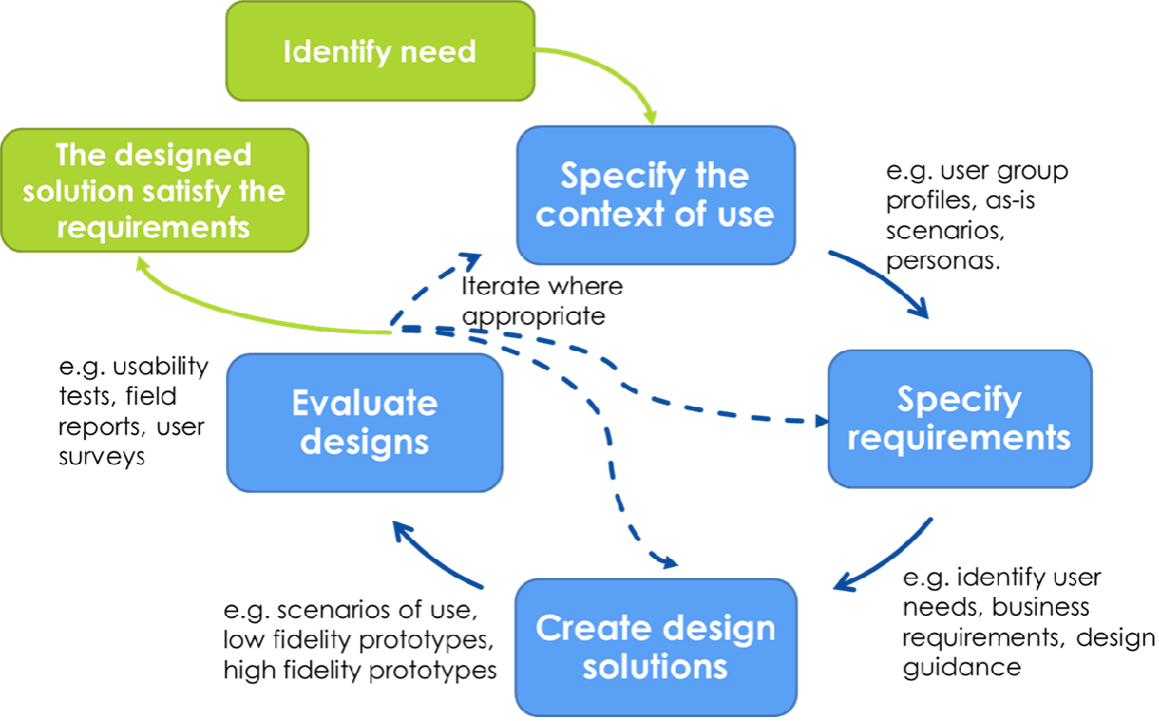
Schematic depicting the phases of the human-centered design process and its iterative nature, as described in the International Organization for Standardization (ISO) 9241-210:2019 standard.

On the basis of evaluations, the design process iterates or progresses to the final product development stage if the requirements are successfully met. A schematic of the HCD process, as described in the ISO 9241-210:2019, is depicted in Figure 1 [41].

Despite the potential impact of implementing ISO 9241-210 in improving AT development and reducing the access gap, Ortiz-Escobar et al [42] found that it is poorly understood and rarely applied in the field of AT for persons who are blind. The review also highlights the lack of methodological rigor in participatory methodologies and the shallow inclusion of users’ points when included. The study reveals that innovation tends to focus on system requirements, neglecting its users.

This paper addresses the knowledge gaps in implementing ISO 9241-210 HCD by reporting on an interdisciplinary action research project that combines applied philosophy with engineering sciences. We aimed to evaluate the feasibility and limitations of applying HCD principles in AT development

## Methods

### Ethical Considerations

The research protocol was approved by the Swiss Federal Institute of Technology Lausanne on research involving humans (056-2021 and 068-2022), the Human Ethics Committee of Universidad del Valle (008-022), Hospital Universitario del Valle (029-2022), Universidad Autónoma de Occidente (01-2021), and Instituto para Niños Ciegos y Sordos del Valle (CEI-2022-02). All procedures were based on fully informed consent and ensured the anonymity of participants. Transportation and refreshments were provided, and each participant received COP 40,000 (approximately US $9) as compensation for their time.

### Location: Pilot Country, Colombia

The vast majority of persons with disabilities live in LMICs, and they are disproportionately represented among the poor. Colombia offers the social and infrastructural diversity needed to enhance the relevance of this study.

Approximately 2 million people in Colombia live with some form of visual disability, representing over 4% of the country’s population [43,44]. In this group, more than 80% live in poverty, and illiteracy rates are 3 times higher among individuals with VI compared to the general population [45].

Colombia is a signatory country to the Convention on the Rights of Persons with Disabilities. Although classified as a middle-income country, its socioeconomic diversity and regional infrastructure variations provide a rich context for drawing insights applicable to a range of low, middle, and high-income settings.

While Colombia has made significant progress in addressing accessibility for persons with disabilities, major challenges persist. Barriers include issues with roads (45%), sidewalks (28%), workplaces (18%), health centers (17%), and schools (15%) [46].

### Testing the HCD

In this work, we implemented the HCD methodology to develop an initial prototype of a navigation AT for individuals with VI. This process followed one full iteration of the 4 design phases outlined in ISO 9241-210:2019 [41], as introduced in the previous section. The following section outlines how each phase of the HCD was operationalized in the context of our project.

Although this work constitutes one complete iteration of the HCD process leading to functional prototypes, its implementation was not linear. Due to significant challenges related to ethics clearance and national legislation (discussed in the results section), we were unable to engage end-users at the outset of the design process as envisioned by HCD principles. Ethics boards required highly detailed and finalized documentation—even for noninvasive, early-stage AT assessments. As a result, the team initiated a preparatory cycle to gather the necessary information for ethics submissions and to ensure continuity in the research and development timeline. This process is summarized in Table 1.

**Table 1:** Summary of the design process and methodologies implemented in this work.

Phase	Preparatory development cycle	Main UCD^a^ cycle
Phase 1: specify the context of use	Literature reviews Internal expertise Informal research interactions Context study^b^	User study (mixed methods approach): Qualitative interviews (WG-SS^c^; 19 participants) Semistructured interviews and focus groups (19 participants) Focus group (5 participants) User feedback on low-fidelity prototypes (19 users) “Wizard of Oz” tests with users (19 users)
Phase 2: specify the user requirements	Analyze data Define initial requirements	Analyze data Refine requirements based on the user study
Phase 3: create design solutions	Design concepts^b^ Refine design concepts using screening matrices and weighted rating techniques Hardware low-fidelity^d^ prototypes Software test protocol^e^	Refine the designs based on updated and validated requirements Functional subsystems fabrication (camera casings, audio processing, etc) Consultations: DPOs^f^ and users Subsystems integration and optimization Functional prototype
Phase 4: evaluate the designs with users	—^g^	Operational tests (head-level object identification, obstacle avoidance, and local navigation; 20 users) Semistructured interviews (20 users)

^a^UCD: user-centered design.

^b^Multiple iterations.

^c^WG-SS: Washington Group Short Set of Questions [47].

^d^Evaluated by users during the user study phase.

^e^Assessed with users in the “Wizard of Oz” test.

^f^DPO: disabled persons organization.

^g^Transition to Main UCD Cycle–User Study.

During this preparatory cycle, initial design concepts were developed using literature reviews, internal technical and social science expertise, and informal research interactions. This adaptive strategy enabled the team to create contextually relevant documentation for ethics approval while simultaneously advancing hardware and software development.

Following ethics clearance, the project entered a formal user engagement stage. Hardware concepts were translated into low-fidelity prototypes, while the intended software interaction commands were structured into a test protocol. These prototypes were presented to users for direct feedback, and the interaction protocol was evaluated through “Wizard of Oz” testing to refine user requirements and system functionalities.

In addition, during the technology development (phase 3), smaller-scale interactions with selected users and disabled persons’ organizations (DPOs) were carried out. These engagements served to confirm key features and refine aspects of the developing functional technology, particularly in terms of usability, interface design, and contextual relevance.

### Context Analysis

We used an action research approach [48] to accompany the technology development, addressing the fact that research into UCD implementation for persons with disabilities, including persons with VI, has largely been overlooked, despite its crucial role in improving well-being. We recruited participants through snowball sampling, facilitated by DPOs, cultural institutions, and rehabilitation centers. This method leveraged existing networks within these communities to identify and refer potential participants who met the study’s criteria. We required eligible participants to be blind or have major VI, be aged ≥18 years, and be available to participate in at least three 1-hour interviews over 18 months. Recruitment continued until data saturation was achieved, ensuring comprehensive coverage of the research themes. To maintain a focused exploration of experiences specific to individuals with VI, participants with any additional disabilities were excluded. A detailed overview of participant characteristics is provided in Multimedia Appendix 2.

The study initially included 19 participants. We implemented a mixed methods approach: after collecting sociodemographic data, qualitative interviews were conducted to gain in-depth insights into individuals’ lived experiences of impairment, their experiences using AT, and their freedoms and limitations. Furthermore, via semistructured interviews and focus groups, we explored how technology can help bridge the gap between the concept of well-being for individuals with visual disabilities and their current lived experiences. This information was used to inform the design phase of the AT project.

Subsequently, a focus group was conducted with 5 (26%) of the 19 participants, and the role of technology in their lives was explored in depth through questions on the following topics: (1) most frequently used ATs, (2) utility of technology, (3) the opportunities gained through it, (4) the desired opportunities, (5) the disadvantages or dangers of using technology, and (6) the characteristics that AT should have to enhance well-being.

All qualitative data were audio-recorded, transcribed, and analyzed using thematic analysis, following the approach outlined by Braun and Clarke [49]. This method supported an in-depth, flexible interpretation of the dataset, allowing for the identification of patterns of meaning across participant accounts. Two authors (LMO-E and MRV) independently coded a subset of transcripts and met regularly to discuss emerging codes and resolve discrepancies, thereby ensuring intercoder reliability. Disagreements were addressed through a consensus-based approach, with a third author (MAC) consulted, when necessary, although this was rarely required. We used strategies such as peer debriefing, triangulation with field notes, and maintaining an audit trail. Member checking was also performed with a subset of participants to verify the credibility of themes. Saturation was monitored throughout data collection and analysis and was considered reached when no new themes emerged from the last 2 interviews.

### “Wizard of Oz” Test

**Figure 2:**
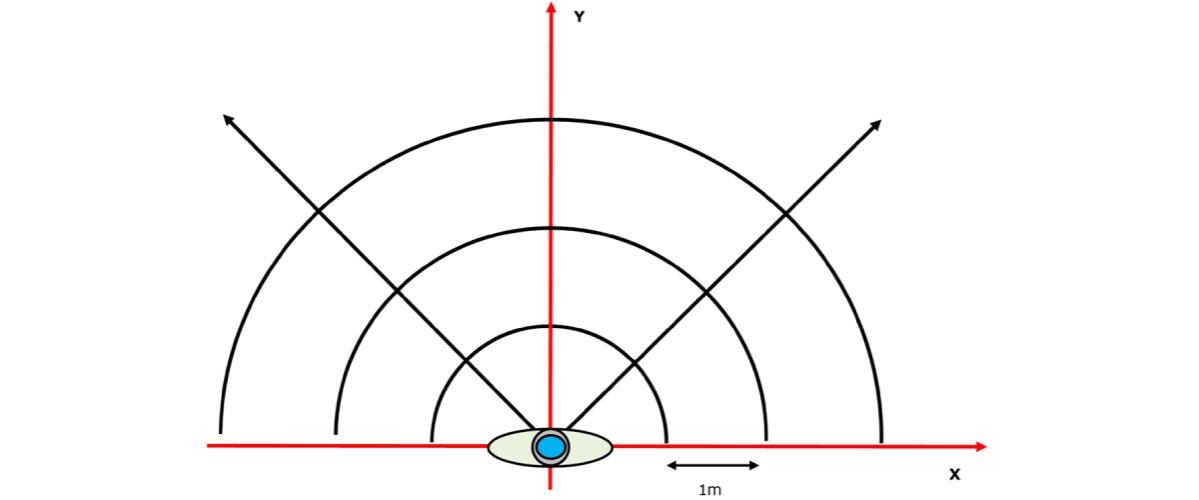
Ground plane view of the test pattern, with the participant with visual impairment positioned at the center of the coordinate frame.

The “Wizard of Oz” method is a well-established technique in human-computer interaction and early-stage product development. In this approach, users interacted with a prototype that appeared autonomous but was controlled by a human operator rather than the computer system [50,51]. This test was conducted with 19 volunteers with VI, using the setup illustrated in Figure 2 [52]. The procedure implemented is described in Textbox 2.

**Procedure implemented during the “Wizard of Oz” test.**

1. The researchers read movement instructions to the participant with visual impairment (VI), simulating the guidance intended to be delivered by the developed navigation system. The participant with VI was exposed to four different types of instructions as follows:
  Walking 1 m or 2 m forward.
  Taking 2 steps in different directions (forward, left, diagonal-left, right, and diagonal-right).
  Moving based on clock-hour directions (eg, 12-, 3-, 9-, 2-, and 10-o’clock positions).
  Walking considering a virtual obstacle located at 1 m forward, left, diagonal-left, right, or diagonal-right.
2. The participant was instructed to perform the movements using their whole body, avoiding any independent torso movement.
3. The participant begins moving, initiating video recording.
4. Upon completing the movement, the video recording is stopped.

This procedure is performed for motions described in 1.1, 1.2, 1.3, and 1.4 and for all participants. For every motion, the participant starts from the same initial position.

The recorded video securely stored and processed using computer vision algorithms specifically developed for this study. The analysis includes measuring the distance traveled and the associated error, as well as assessing the orientation error using 2 predefined orientation methods.

### Prototype Evaluation With Users

We conducted a user-perspective study with 20 participants. We initially invited those who participated in the first phase of the study, of whom 13 were available, and then recruited an additional 7 participants using the same inclusion criteria as in the first user study. We aimed to gather a comprehensive, user-centered perspective on the acceptability, appropriateness, ergonomics, and feasibility of the proposed assistive navigation system for individuals with VI.

**Figure 3:**
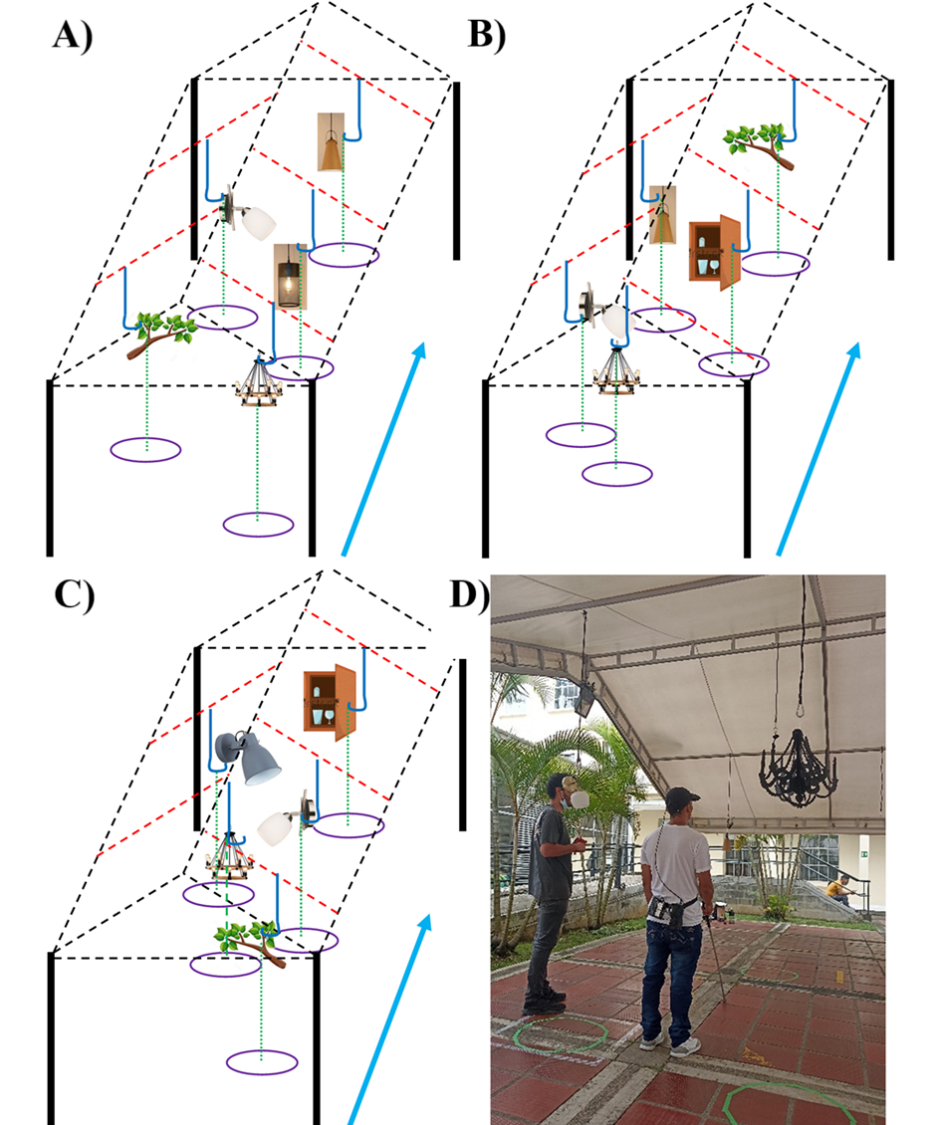
(A) Object configuration for the head-level obstacle identification test; (B) object configuration for the obstacle avoidance test; (C) object configuration for local navigation tests; and (D) photograph of the user perspective study test venue.

The evaluation was conducted indoors in a controlled 6×9 m² test area covered by a tent (refer to Figure 3D). Each participant underwent a 2-hour session consisting of 3 operational tests—head-level object identification, obstacle avoidance, and local navigation—while wearing a selected design variant of the prototype. These tests were structured to validate the system’s core functionalities (Textbox 3 [52]).

The evaluation also included a session in which each participant tried on and rated all available design variants—glasses, a cap, and a necklace—concluding with a semistructured interview.

To ensure comparability, all operational tests were conducted using the same prototype variant: the cap, which had emerged as the preferred option in earlier evaluations. Although the glasses variant was appreciated for its aesthetics and integration, it was the least favored due to its weight and the resulting pressure on the nose. This feedback will inform future ergonomic refinements.

After each test, survey responses were collected, and all system data, including video streams, object recognition inferences, point clouds, and audio feedback logs, were stored in ROS bag format for subsequent analysis. Data collection followed a standardized file-naming convention, including user ID, date, time, and test name.

Each participant completed all 3 test scenarios in a randomized order to reduce learning effects. To further prevent participants from memorizing obstacle locations, the object configurations were altered between tests. The tests were conducted with the support of a companion and a test engineer, following a structured protocol to ensure safety and consistency.

**Operational tests for prototype evaluation with users.**

- Object identification: participants were guided to specific positions where the system described, via audio feedback, nearby head-level objects, such as tree branches or chandeliers (Figure 3A).
- Obstacle avoidance: participants walked through the space while receiving audio cues to detect and avoid obstacles (Figure 3B).
- Local navigation: participants were guided along a suggested path using real-time audio instructions to navigate around head-level obstacles (Figure 3C [52]).

## Results

### Overview

This section summarizes the results of each phase of the HCD process implementation for developing a navigation system for individuals with VI, as described in the Methods section.

The project involved researchers from Colombia and Switzerland, with fieldwork conducted in Colombia. Before initiating the user and context study intended to guide the technology development, we secured support from several local DPOs. Each partner institution required clearance from its in-house ethics committee, often involving significantly different and uncoordinated processes, a common challenge, particularly in fragile research ecosystems.

A key finding concerns inadequate ethics clearance and legislation. Legislation and respective ethics committees in both countries mandated detailed, finalized research plans, even for assessing and approving noninvasive Class 1 medical devices with low-fidelity prototypes. Consequently, the research team could not formally involve end users until ethics clearance was obtained. This requirement led the team to propose a design based on literature reviews, internal expertise, and informal research interactions rather than the originally planned empirical research. Therefore, pursuing a linear application of the ISO principles proved impossible, a common observation in today’s AT development literature [42]. Nonetheless, to align as much as possible with HCD principles, we conducted empirical research to identify user priorities after obtaining all ethics clearances, revealing several limitations as outlined in this section.

### Phase 1: Specify the Context of Use

#### Findings on Perceptions and Personal Experiences

Our findings reveal a disconnect between available technology, such as ETAs, navigation apps, and ultrasound canes, and the users’ needs and realities. Available products were of little or no use in the average urban environment due to damaged infrastructure, uneven terrain, or the complete absence of sidewalks in some areas. This limited utility persisted even in better-served areas due to governance and maintenance issues. For instance, traffic rules were frequently disregarded, rendering streets and available infrastructure, such as tactile paving, ineffective for persons with VI. In the social and personal sphere, interviews uncovered that few AT devices, such as the AuxDeco system and ultrasound canes, were available to persons who are blind via international donations to DPOs.

All users expressed dissatisfaction with the available technology, deeming it inadequate. Reasons cited for the nonadoption of the technology included (1) information saturation with irrelevant data, (2) difficulty and discomfort in use, and (3) drawing unwanted attention, making them feel different and excluded, akin to being labeled as “Robocop,” as mentioned by many. These findings indicate that high prices are only one facet of the problem; even if the cost barrier is overcome, the devices fail to meet the users’ needs. Moreover, the results unveil complex and concealed barriers faced by individuals who are blind, such as multidimensional poverty, discrimination, experiences of violence, and the association of visible technology with further stigmatization. Aside from dislike, users stated that using large, visible technology on the street exposes them to theft. They described cases of friends who have had their cell phones stolen and even shared experiences about their daily efforts to camouflage their technology use to avoid being robbed. This quote summarizes security risks linked to using technology in the streets:

I had a phone stolen because I was listening to the app to find out which route was coming.

Participant 15A

The complete results reporting on users’ experiences and expectations on AT are reported in a previous study by Chavarria Varon et al [15].

#### Findings on Priorities, Opportunities, and Constraints

Participants prioritized technology that supported social connection and enabled greater bodily mobility. They emphasized that technologies that create social distance, by disrupting communication, obstructing physical interaction, or attracting unwanted attention and stigma, were considered unfit for purpose. Movement was a recurring theme in their narratives, with many expressing their frustration over limited freedom and the inability to fully experience their bodies in motion. Participants longed to engage in motor activities, such as running, cycling, dancing, and skating, often associating speed with a sense of vitality and freedom. Although many moved cautiously to avoid injury, there was a strong desire to reclaim and liberate their bodies through active movement, as evidenced in the following quote:

I would like to run...I can run, but I mean...going down some stairs fuuufff fast. That’s something I can’t do now. Running...or swimming. I can’t swim in a river, I’m afraid of drowning.

Participant 6A

Most participants emphasized their desire for a device that is minimally invasive, enabling them to preserve their identity while using it. They stressed the importance of integration, minimal expression, and “street-proof” design to mitigate concerns about potential damage or theft due to its visibility.

Regarding the drawbacks and risks associated with technology, 2 main points were mentioned. First, participants expressed concerns about becoming overly reliant on technology. Second, they raised concerns about increased security risks, particularly in the local context. They noted that the more they relied on mobility-related applications, the less they exercised their orientation and mobility skills.

Regarding navigation in urban environments, some participants reported the condition of the streets as a difficulty for mobility. In some cases, the streets are unpaved, the terrain is uneven, and there are no marked areas for pedestrian traffic. Others noted that the sidewalks in the city are often occupied by cars, motorcycles, and sales stands, among others. These obstacles could be detected by properly using a cane. However, high obstacles, such as lattices, signs, fences, tree branches, umbrellas of street vendors, exterior snail-shaped stands, or the tops of fences, cannot be detected. These are just some of the objects with which participants reported injuring themselves while walking through the city.

To navigate more smoothly, several participants reported stepping off the sidewalk and walking along the edge of the road, despite the risk of being hit by a vehicle. While this strategy helps them avoid certain obstacles, it does not eliminate the risk of injury. For example, 2 participants reported damaging their glasses after colliding with the rear of a parked van. Such incidents typically occur on smaller streets but have occasionally been reported on main avenues as well.

#### Findings on Technology Implementation

The context study revealed a major gap between prototype development and market adoption of assistive and rehabilitation technologies for persons with disabilities in Colombia, an issue common in many LMICs. Most devices remain at the laboratory stage, with very few achieving local manufacturing or regulatory approval. Key barriers include weak collaboration among stakeholders, limited regulatory and commercialization expertise, and a lack of business focus in research. As a result, Colombia remains heavily reliant on imported technologies, which drives up costs and limits access. Strengthening local manufacturing could improve affordability and contextual relevance. Detailed results and analysis are presented in a previous study by González-Vargas et al [8].

### Phase 2: Specify Requirements

#### Findings on User Requirements

##### Obstacle Detection and Avoidance

The AT should efficiently and promptly detect obstacles, guiding the user along a clear path to avoid them. The system should prioritize detecting high obstacles not detectable by the white cane.

##### User Comfort

All portable components should be compact, inconspicuous, and lightweight for prolonged wear.

##### Adaptation to Socioeconomic Context

To develop a solution applicable in numerous communities worldwide, the system should have the characteristics described in Textbox 4.

**Desired characteristics of assistive technologies (ATs).**

**Not depend on a continuous internet connection**

- Assistive devices reliant on constant internet and GPS connection are impractical in rural areas or low-income neighborhoods with poor connectivity. Thus, the system must operate locally (not in the “cloud” or external servers) to function in locations lacking high-speed internet or GPS-denied environments.

**Not put the user at risk**

- All components must be easily concealable to reduce theft risk.

**Be economically sustainable**

- The AT’s total cost should be less than 10 times that of similar market solutions. It should also use locally accessible materials and components and be easily maintainable.

**Robust in tropical conditions**

- The ATs should be able to withstand high temperatures and humidity.

**Maintain autonomy**

- The device should maintain prolonged energy autonomy.

##### Ease of Use

The device should feature an intuitive user interface that is easy to learn and operates independently. Audio interfaces are preferred over tactile ones for faster information acquisition without occupying the hands. In addition, the output audio interface should not impair users’ hearing capacity.

##### Information Presentation

Data should be presented in a straightforward, concise, and easily understandable manner, aligning with findings from the “Wizard of Oz” test.

##### Aesthetics

Portable components should be visually appealing, with electronics easily concealable. Anything visually conspicuous, such as large electronic devices on their face, may be rejected. The design should be “aesthetically intelligent,” skillfully harmonizing sensory attributes, contextual nuances, cultural significance, and functional effectiveness to craft products that evoke a greater sense of empathy from users [53].

#### Findings on the “Wizard of Oz” Test Outcomes

**Figure 4:**
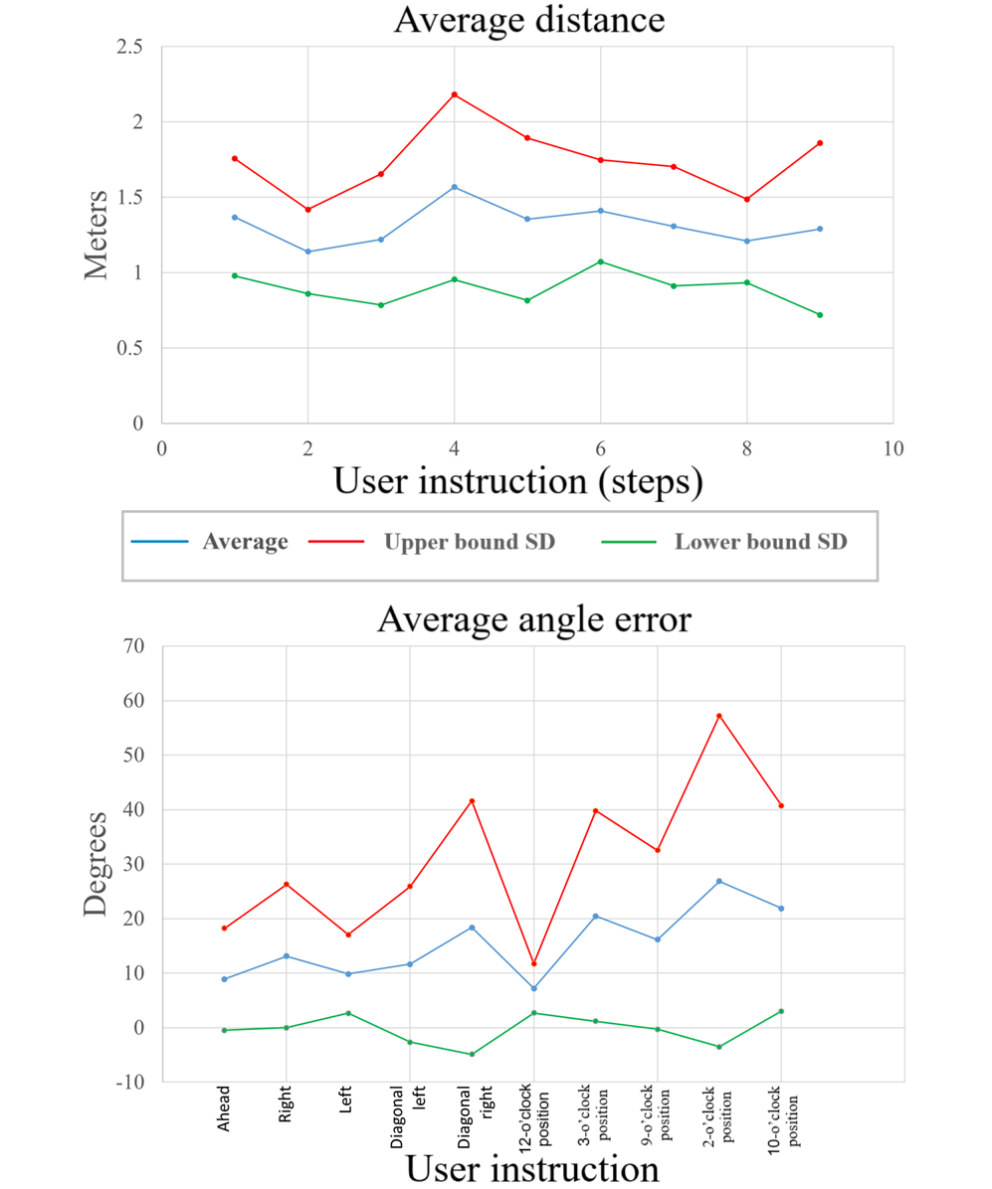
Quantitative results of the “Wizard of Oz” tests. Specifically, the distance and angle average error.

All the videos recorded in the “Wizard of Oz” tests were digitally processed to measure the distance, angle, and trajectory performed by the participants with VI (Textbox 5). Figure 4 presents the average distance traveled and the angular error for participants following the user instructions. The blue line represents the average, while the red and green lines indicate the upper and lower bounds of the SD.

**Outcomes of the “Wizard of Oz” tests.**

- Distance traveled: participants were instructed to move 2 steps in different directions, resulting in an average movement of 1.32 m, which aligns well with the calculation in Equation (1).
- Angular error: the angular error was measured relative to ideal orientation values (0°, 45°, 90°, 135°, and 180°). The first 5 orientation instructions (“go ahead,” “to the right,” “to the left,” “diagonal left,” and “diagonal right”) demonstrated less confusion for participants compared to instructions using clock references (eg, 10-, 3-, and 12-o’clock positions).

**Figure 5:**
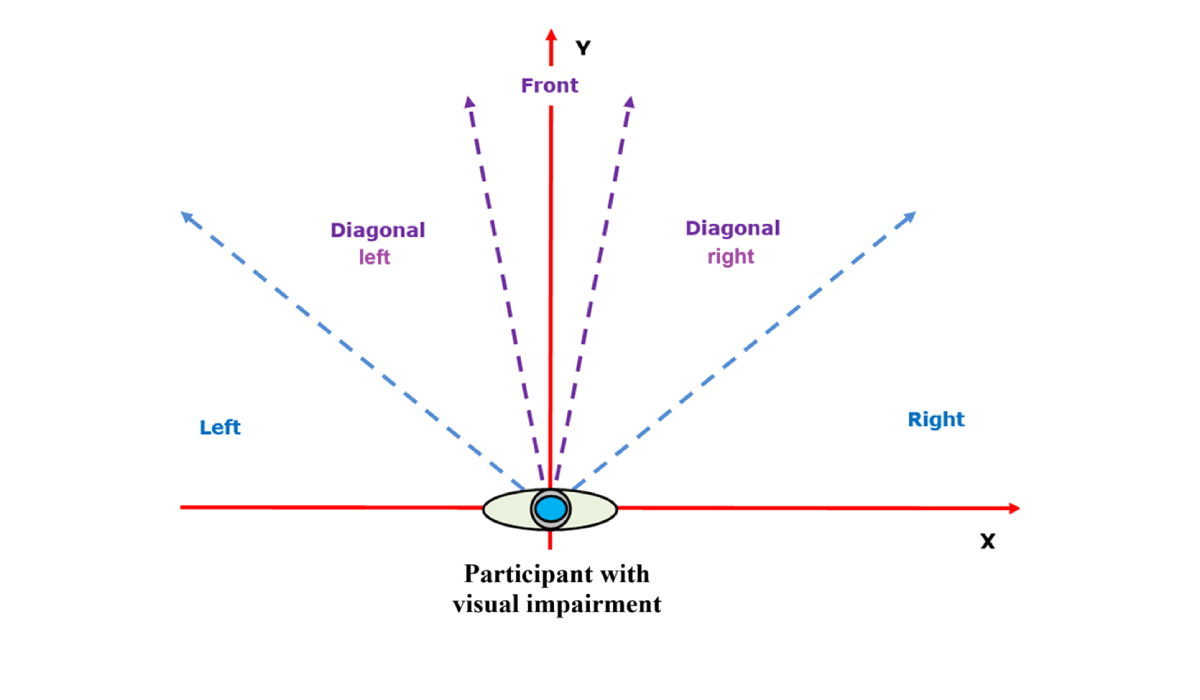
Preferred orientation cues for individuals with visual impairment based on the “Wizard of Oz” test results.

Figure 4 illustrates the average angle error for the 1.1 and 1.2 intersection subtests of the “Wizard of Oz” tests, as described in the Methods section. On the basis of the analysis of these results and the literature review, it can be concluded that the most effective way to provide distance and orientation information to users is through step counts and directional instructions (left or right, diagonal left or right, and front or back), as shown in Figure 5 [52]. Notably, clock-based directional instructions exhibit, on average, 50% more error compared to left or right, diagonal left or right, and front or back instructions. This angular error analysis supports the selection of the orientation instructions listed in Table 2, where orientation is converted from degrees to descriptive instructions.

The relationship between the range to the detected object and the number of steps required is defined by Winter [54]:



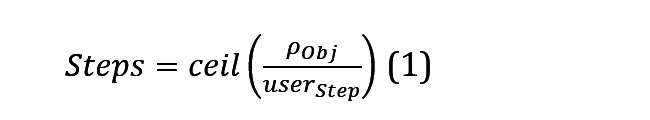



Where *ρ_obj_* is the range to the detected object and *user_Step_* is the anthropometric measure of one step (in meters) based on the user’s height.

**Table 2:** Conversion table for orientation instructions.

Range (degrees)	Descriptive instruction
<50	Right
50-75	Diagonal right
75-105	Front
105-130	Diagonal left
>130	Left

### Phase 3: Create the Design Solutions (Technology Development)

#### Overview

**Figure 6:**
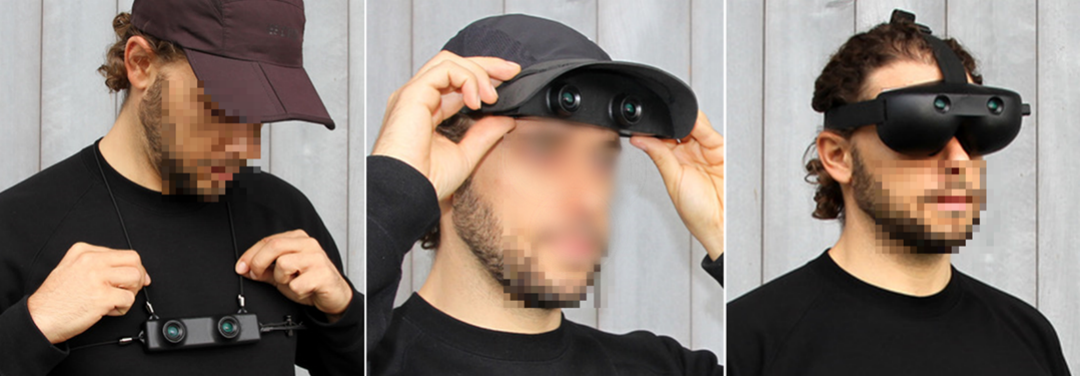
Images of the manufactured casings for the functional prototypes.

**Figure 7:**
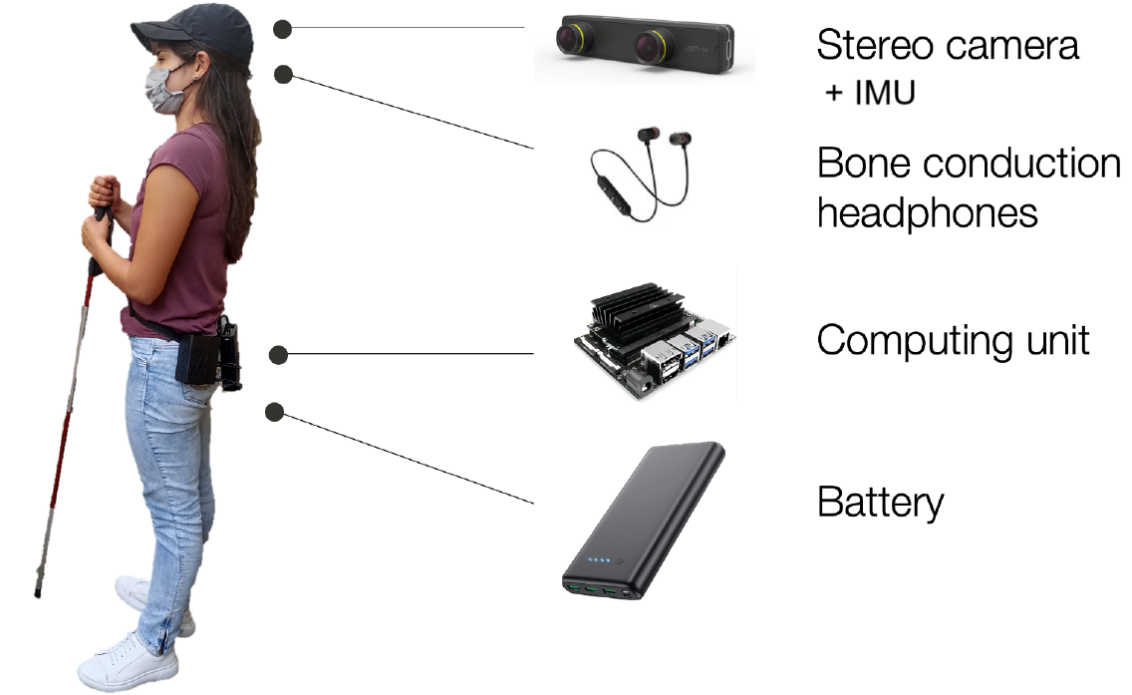
Electronic hardware devices and their user-mounted positions in the proposed navigation system. IMU: inertial measurement unit.

During the prototyping stage, user requirements defined in phase 2 were translated into measurable specifications, which guided the functional decomposition and benchmarking of key performance metrics. Following the design methodology proposed by Ulrich and Eppinger [55], we defined objective specifications, explored design alternatives using screening matrices, and iteratively refined the most promising concepts through early usability tests. Low-fidelity design variants were crafted and reviewed by the team and the users, leading to the selection and 3D printing of 3 wearable casing prototypes—glasses, a cap, and a necklace (Figure 6). To expedite early testing, the first functional prototype used off-the-shelf electronic components, enabling the collection of valuable user feedback in the early phases of the technology development without overcommitting time and resources to the development of customized electronics. Figure 7 illustrates the electronic components and their placement on the user’s body. The ZED Mini stereo camera (Stereolabs), connected via USB and equipped with an integrated inertial measurement unit (IMU), serves as the primary sensor. Data are processed by the Jetson Nano computing unit, which identifies head-level objects and delivers audio feedback via Bluetooth headphones (9 DIGITAL bone conduction Bluetooth headphones). The IMU helps filter frames not captured from the forward view. A battery pack powers the system, with the camera embedded in wearable accessories for aesthetic, comfort, and safety, while other components are carried on a waist belt.

#### Software Development

Addressing the user requirements in phase 2, and considering technical feasibility, and the necessity for the prototype device to be suitable for LMICs, the technical specifications were defined (Textbox 6), aligning with the rational unified process methodology [56].

**Figure 8:**
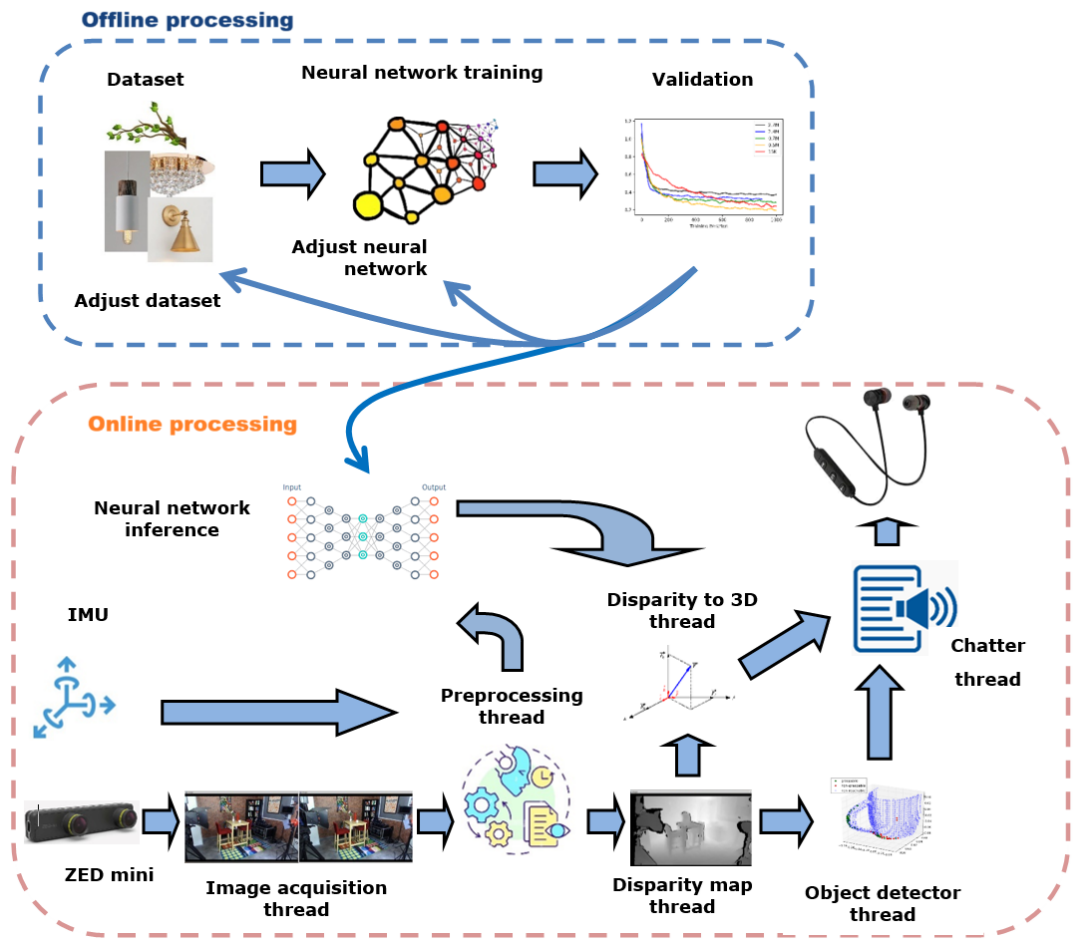
Conceptual diagram of the navigation prototype. The top section (blue border) shows the offline training phase, whereas the bottom section (red border) represents the online inference phase. IMU: inertial measurement unit.

Figure 8 illustrates the software architecture of the prototype, which operates in 2 stages: an offline stage for training a deep neural network, and an stage for real-time detection and classification of head-level obstacles using an optimized model deployed on a Jetson Nano.

Stereo images are preprocessed to enhance quality; blurred images and those captured while the user is looking down (based on IMU data) are discarded. The cleaned images are processed by 2 components: the neural network inference node, which outputs object classifications and bounding boxes, and the disparity map node (based on Isaac ROS Image Pipeline [57]), which produces depth information.

**Technical specifications of the prototype device.**

- Perception data comprises stereo image and inertial measurement unit (IMU) data.
- Preprocessing improves image acquisition by excluding frames captured from nonforward perspectives using IMU data.
- The system will compute the relative distance between users and the nearest object using a disparity map obtained by processing stereo image pairs.
- The system will conduct the detection and classification of head-level objects, such as ceiling lamps, sconces, tree branches, and chandeliers.
- Other objects not classified by the neural network model will be detected as obstacles.
- The system will combine the detection results and the distance to object or obstacle estimates to generate audio alerts for users.

These outputs feed into the disparity-to-3D node, which calculates the object’s distance and orientation relative to the user. If the neural network fails to classify an obstacle, the object detector node uses the 3D point cloud to detect and segment unclassified but potentially dangerous objects. In both cases, distance and orientation are translated into navigation instructions. The prototype uses the disparity-to-3D node for path planning and the object detector node for obstacle detection. A comprehensive description of the software development process and system features is presented in the study by Muñoz et al [52].

#### Findings on Audio Interface

The audio interface integrates into the system software through the chatter node (Figure 8), which receives information from the 2 system operation modes (path planning and object detection modes) and transforms it into audio output.

In the path planning mode, the input includes planned path directions from the system, generating corresponding audio instructions, such as “walk to the right diagonal,” “walk straight ahead,” and “stop.” Alternatively, the system can provide the user with the number of steps required for the suggested path

In object detection mode, there are 2 inputs. The first comes from object detection by the deep neural model, you only look once (version 5), providing details about the detected object’s classification, direction, and distance in steps from the user’s position. An example of audio output is “Tree branch two steps to the right.” The second input is obtained from local map detection, providing information about the distance (in steps) and direction of an unclassified object, for example, “Object two steps to the left diagonal.” In addition, the chatter node generates extra audio warnings to enhance user-device interaction, including messages such as “low battery,” “device shutting down,” among others.

Navigation instructions are delivered to the user via a bone conduction headphone, which was chosen due to significant advantages, particularly for enhancing safety, improving spatial awareness, and facilitating a more intuitive navigation experience. One of the primary benefits of bone conduction headphones is their ability to transmit audio instructions while keeping the user’s ears open to ambient sounds. This feature is crucial for individuals with VI who rely on auditory cues from their environment to navigate safely. Studies have shown that traditional headphones can obstruct environmental sounds, which may lead to dangerous situations, as users may not hear approaching vehicles or other hazards [58,59]. By contrast, bone conduction technology allows users to receive navigational audio cues while remaining aware of their surroundings, thereby enhancing their overall situational awareness and safety during navigation [21,60].

### Phase 4: Evaluation With Users

#### Findings on Device Evaluation With Users

After analyzing the results of these tests, the object recognition achieved a 91% success rate on average. The risk of collision in obstacle-avoidance mode was low, with 80% categorized as low risk and 10% as medium risk. In the local navigation mode, the risk of collision was slightly higher, with 74% categorized as low risk and 13% as medium risk. Overall, users reported feeling comfortable with the system and quickly learned how to use it.

Regarding design prototypes (Figure 6), users ranked the harness or necklace first (12/20, 60% of users), the cap second (13/20, 65% of users), and the glasses last. The main reason for this choice was comfort, whereas many participants mentioned that the designs were not discreet enough.

The interview questions were designed based on the preliminary analysis of the initial data collection round, considering the capabilities approach and the functions of the assistive device prototype. Participants’ answers formed the basis for developing a focus group with 6 participants.

The results of the ergonomics survey support this statement, revealing different levels of satisfaction among participants regarding different aspects of the devices. This disparity is particularly evident when examining respondents’ comments for each item and observing the relationship between their answers and their needs and preferences. For instance, individuals blind from birth may prefer a device that does not obstruct their ears, while participants with residual visual abilities may find glasses obstructive.

Despite these differences, common preferences emerged, including a desire for improvements in the size, discretion, and aesthetics of the device. Participants expressed a preference for a smaller, inconspicuous, wireless device that does not interfere with their attire or activities nor reduce their body freedom, that is, occupy at most one part of their body. Also, the device should not put their life or bodily integrity at risk, or make them feel like outsiders, and it should allow them to be part of what they value. Participants expressed the importance of having choices, which is evident in their responses regarding color preferences. Those who desired the device to be available in a different color avoided asking for just one option and instead sought a variety of alternatives from which interested individuals could choose. Finally, the motivation for using this type of AT revolves around reinforcing autonomy and safety while moving around.

## Discussion

### Principal Findings

This paper reports on the feasibility and limitations of applying HCD principles in AT development (ISO 9241-210:2019 standard) [41]. Despite the clear benefits and good intentions of the HCD principles, we encountered substantial barriers to their implementation in the technology design due to a mismatch between available research infrastructure, methodologies, and legislative frameworks. Nevertheless, notwithstanding these shortcomings, our initial prototype demonstrates significant progress in meeting user expectations and adapting to the local context, distinguishing itself from existing ETAs in both the market and literature. We used rigorous qualitative methodologies to document users’ experiences and interactions with technical features and expectations regarding AT beyond this navigation tool. In terms of improving available innovation in AT, our prototype offers key features, such as an unobtrusive interface, clear instructions, and semiconcealed electronic components, contributing to its appeal, and significantly lower cost than similar solutions (5 to 15 times lower than similar products).

One of the main challenges in applying the HCD methodology is the misfit between our research needs and available legislative infrastructure. We prioritize user involvement in our research proposal, aiming for meaningful engagement in the conception, design, and testing. However, researchers cannot engage with end users until they secure ethics clearance, which involves submitting a comprehensive and finalized research plan for evaluation and approval. Consequently, most decisions must be made before any direct interaction with users occurs, allowing little flexibility for significant design changes. This challenge is compounded by institutional bureaucracy, for example, in this project, the research team had to apply to 6 different ethics committees, each with its own procedures and legal frameworks. This results in limited user involvement and, therefore, a constrained application of the HCD approach.

While commendable progress has been made in ensuring that technological research respects the rule of law and the rights of its potential final users, particularly those at risk of vulnerability, such as persons with disabilities, our results indicate that more needs to be done to ensure that individuals are protected from harm and coercion, thus having the opportunity to safely contribute if they wish to innovate and research. This would allow AT developers to improve the design process and have a better chance of closing the existing AT gap. The balance between safety needs and the demands of final users and innovation research needs to be reviewed and better aligned to move forward.

In addition, while ISO 9241-210:2019 guides usability and user experience, it does not delve deeply into the technical intricacies of implementation, particularly in contexts involving mechatronic systems. In such cases, additional standards specific to hardware and software engineering become necessary to ensure comprehensive and effective development practices. In our case, we used the rational unified process methodology to obtain low-level technical requirements and foster active stakeholder participation throughout the development life cycle. Hence, while ISO 9241-210:2019 provides a solid foundation for UCD, it should be supplemented with other standards and guidelines addressing the technical complexities inherent in AT solutions incorporating both hardware and software components. This holistic approach ensures optimization of both the user experience and the technical functionality of the product for ultimate success.

This is just the first iteration of the HCD methodology, and several aspects remain to be enhanced in future redesigns. Feedback from functional tests identifies areas for improvement, including reduced system size and weight, enhancing aesthetics, and improving the concealability of components. Furthermore, integrating features such as currency detection and potholes warnings would further enhance usability and better meet user needs.

Future iterations will also involve testing the device in different Colombian cities to assess its adaptability across varied urban environments. Expanding trials to other countries and continents is envisioned in later phases, ensuring that contextual variations are considered in the development of inclusive and adaptable solutions.

Moreover, a study on legal regulations and ethical risk frameworks governing user involvement in AT development will be proposed. This will aim to establish clearer operational guidelines for navigating regulatory and ethical challenges, ultimately facilitating the safe and efficient participation of persons with disabilities in shaping the technologies they will use. Strengthening governance structures, including ethics committees, through a well-defined risk assessment approach will be key to advancing this process.

Looking ahead, sustained collaboration with end users and iterative design refinement through HCD methodology will be imperative in developing ETA solutions that fully meet the diverse needs of individuals with VI, particularly in LMICs.

Cross-fertilization of knowledge is key to addressing changes related to AT. Thus, multidisciplinary research requires significant work, commitment, and compromise, as each scientific discipline works under different scientific paradigms, which can lead to conflicts of interest. To ensure its implementation, multidisciplinarity shall be perceived as a common aim and accompanied by a work plan, as is done for other research goals. This project shows that multidisciplinary teams can lead to generating novel combinations and added-value technologies.

Finally, considering the conducted user tests, a common question was “Were you able to fully understand the audio instructions?” The percentage of users who responded positively increased over the course of the tests, with 85% (17/20), 90% (18/20), and 100% (20/20), respectively. This indicates that users gradually became more accustomed to the device. Another common question was “How confident are you to avoid more obstacles if you would repeat the test?” The percentage of participants who reported feeling confident or very confident also grew, from 60% (12/20) to 70% (14/20), and then to 90% (18/20). This demonstrates an increase in user confidence with continued use of the device.

### Conclusions

This paper explores the design and development of a context-sensitive, person-centered navigation device, guided by HCD principles in accordance with ISO 9241-210:2019 standard. Despite significant challenges, the development process has yielded promising results, demonstrating notable advancements in meeting user expectations and adapting to local contexts at a notably reduced cost compared to existing alternatives, showcasing the potential of user-centered innovation in AT. However, legislative and methodological barriers, including limited flexibility for iterative design changes, significantly constrained the application of HCD principles. These limitations highlight the need for a more cohesive approach that aligns research methodologies, technology development practices, and regulatory frameworks to maximize the benefits of HCD.

Despite the decades-long promotion of HCD, its rigorous and systematic application remains elusive in the AT domain, particularly for individuals with VI [15-18,42]. A review of existing literature and practices reveals a lack of empirical evidence and methodological rigor in many user-centered-design frameworks [15-17,42,61]. Frequently, researchers mislabel informal or minimal user interactions as sufficient to qualify their developments as HCD, which is both ignorant and unethical [42]. Adherence to ISO standards remains inconsistent, with many projects failing to meaningfully engage users throughout the development life cycle. During the practical application of HCD in our work, 3 major barriers to its effective implementation were identified, as detailed in Textbox 7.

**Barriers to the implementation of human-centered design (HCD) principles.**

**Lack of systematization**

- The absence of standardized processes for integrating HCD into assistive technology (AT) development often results in superficial or late-stage user involvement. Tools and methodologies for systematic engagement are inadequate, and ethics protocols frequently impose rigid, predefined research plans that hinder iterative design changes. While standards provided by the International Organization for Standardization provide a framework, they lack specificity for complex systems, such as mechatronic devices, necessitating additional technical standards for hardware and software integration.

**Regulatory constraints**

- Legal and ethical frameworks, although designed to protect users, can unintentionally restrict their engagement in research. Ethics committees often require finalized research plans before allowing user interaction, leaving little room for early-stage input or midcourse corrections. These lengthy bureaucratic processes create delays, especially in multinational projects, and may isolate researchers from the users they aim to serve.

**Contextual misalignment**

- Assumptions that solutions designed for individuals without disabilities will work equally for individuals with disabilities often lead to suboptimal outcomes. This is particularly true in low- and middle-income countries, where additional contextual barriers must be addressed to ensure the relevance and utility of AT solutions.

To address these challenges, a more flexible, standardized, and user-driven approach to AT development is essential. Sustained collaboration with end users, iterative refinements, and multidisciplinary research are critical for driving innovation and creating effective, inclusive technologies. By fostering meaningful user engagement and adapting methodologies to real-world contexts, AT development can better align with the diverse needs of users, particularly those in underserved regions.

## Appendix

Multimedia Appendix 1 Comparison of the analyzed systems for the development of electronic travel aids, including the system developed in this work for reference.

Multimedia Appendix 2 Sociodemographic data of the participants.

## Abbreviations

AT: assistive technology
DPO: disabled persons organization
ETA: electronic travel aid
HCD: human-centered design
IMU: inertial measurement unit
ISO: International Organization for Standardization
LMIC: low- and middle-income country
RGBD: red, green, and blue, plus depth
VI: visual impairment
