# Supplementary material for: Challenges and Opportunities of the Human-Centered Design Approach: Case Study Development of an Assistive Device for the Navigation of Persons With Visual Impairment
Source: JMIR Rehabil Assist Technol. 2025 Aug 18;12:e70694. doi: 10.2196/70694 (PMC12402732; doi:10.2196/70694)
Supplement: Multimedia Appendix 1 [file rehab_v12i1e70694_app1.pdf]

Appendix 1: Comparison of the analyzed systems for the development of electronic travel aids, including the system developed in this work for reference.

Reference	Setting	Time	Distance	Dynamic/ Static Obstacles	Sensors	CPU platform	Object recognition	Navigation	CI algorithm
[23]	Indoor	Day	Medimum	SO / DO	RGBD	Google Tango	LO	Ok	Semantic mapping
[24]	Indoor	Day	Medimum	SO / DO	RGBD	Embedded	LO	Ok	Cloud point proc
[25]	Indoor	Day	Medimum	SO	RGBD	Laptop	HO / LO	Ok	Prog hough
[26]	Indoor	Day	Large	SO / DO	LRF and monocular	Laptop	HO / LO	Ok	Fuzzy logic
[27]	Indoor/Outdoor	Day	Medimum	SO / DO	Monocular	Smart phone	LO	Ok	BOW and SVM
[28]	Indoor/Outdoor	Day	Short	SO	Monocular	MCU	No	No	Roving net
[29]	Indoor/Outdoor	Day	Medimum	SO / DO	Ultrasonic and monocular	Smart phone	HO / LO	Ok	SVM
[30]	Indoor	Day	Medimum	SO / DO	Dynamic vision sensor	Embedded	HO / LO	No	Stereo img proc
[31]	Indoor	Day	Large	SO / DO	IP cameras	Smart phone	LO	Ok	No
[32]	Indoor/Outdoor	Day	Medimum	SO	Google glass and smart cane	Embedded	LO	No	YoloV3
[33]	Indoor	Day	Short	SO	Camera, sonar, and button	Embedded	LO	No	MobileNet V2
[34]	Indoor/Outdoor	Day	Medimum	SO	Stereo camera	Embedded	LO	No	MobileNet V1
[35]	Indoor/Outdoor	Day	Short	SO / DO	Camera	Embedded	LO	Ok	MobileNet
[36]	Indoor/Outdoor	Day	Medimum	SO	Camera	Embedded	LO	No	E-BiSeNet
[37]	Indoor/Outdoor	Day	Medimum	SO	Stereo camera	Embedded	LO	No	Yolo V4
[38]	Indoor/Outdoor	Day	Short	SO	Camera, light, sonar, and humidity	Embedded	LO	No	No
This work	Indoor/Outdoor	Day	Medimum	SO	Stereo camera and IMU	Embedded	HO	Ok	Yolo V5
