# Supplementary material for: Challenges and Opportunities of the Human-Centered Design Approach: Case Study Development of an Assistive Device for the Navigation of Persons With Visual Impairment
Source: JMIR Rehabil Assist Technol. 2025 Aug 18;12:e70694. doi: 10.2196/70694 (PMC12402732; doi:10.2196/70694)
Supplement: Multimedia Appendix 2 [file rehab_v12i1e70694_app2.pdf]

Appendix 2: Sociodemographic Data of Participants

Id	Sex	Age	Level of education	Level of education	Self-reported ethnicity	Participation
1A	M	18	Primary	Primary	None	PI
2A	F	31	HE	HE	None	PI & PIV*
3A	F	21	HE(IP)	HE(IP)	None	PI & PIV*
4A	F	28	Secondary	Secondary	None	PI
5A	F	56	NFE	NFE	None	PI
6A	M	23	Secondary	Secondary	None	PI & PIV
7A	M	30	HE(IP)	HE(IP)	None	PI & PIV
8A	M	47	HE	HE	Afro-Colombian	PI & PIV*
9A	M	26	Secondary	Secondary	None	PI & PIV
10A	M	24	HE	HE	None	PI
11A	F	21	HE(IP)	HE(IP)	None	PI & PIV
12A	M	51	Secondary	Secondary	None	PI & PIV
13A	M	29	Secondary	Secondary	None	PI & PIV
14A	M	37	HE	HE	None	PI & PIV*
15A	M	40	HE	HE	None	PI
16A	M	39	HE	HE	None	PI & PIV*
17A	M	24	Secondary	Secondary	Afro-Colombian	PI & PIV
18A	M	19	HE(IP)	HE(IP)	None	PI
19A	F	29	HE(IP)	HE(IP)	None	PI & PIV
20B	F	30	HE	HE	None	PIV
21B	F	28	HE	HE	Afro-Colombian	PIV
22B	F	27	HE	HE	None	PIV
23B	F	25	HE	HE	Afro-Colombian	PIV
24B	M	42	HE	HE	None	PIV
25B	F	49	HE	HE	None	PIV
26B	M	40	Secondary	Secondary	Indigenous	PIV

[A] F: female, [B] M: male, [C] HE: higher education, [D] HE (IP): Higher Education in progress, [E] NFE: No formal education, [F] PI: Phase I, [G] PIV: Phase IV, [H] PI&PIV: Phase I and Phase IV, [I]: *Participation in focus group
